# Inhibition of actin polymerization in the NAc shell inhibits morphine-induced CPP by disrupting its reconsolidation

**DOI:** 10.1038/srep16283

**Published:** 2015-11-05

**Authors:** Gongying Li, Yanmei Wang, Min Yan, Yunshuai Xu, Xiuli Song, Qingqing Li, Jinxiang Zhang, Hongxia Ma, Yili Wu

**Affiliations:** 1 Department of Psychiatry, Jining Medical University, 16 Hehua Rd, Taibaihu New District, Jining, Shandong, 272067, China; 2 Department of Traumatology, the First People’s Hospital of Jining City, Jining, Shandong, 272011, China; 3 Department of Psychiatry, the Second Affiliated Hospital of Jining Medical University, Jining, Shandong, 272051, China; 4 Department of Psychiatry, Binzhou Medical University, Yantai, Shandong, 264003, China; 5 Department of Nursing, Jining Medical University, Jining, Shandong, 272067, China

## Abstract

Drug-associated contextual cues contribute to drug craving and relapse after abstinence, which is a major challenge to drug addiction treatment. Previous studies showed that disrupting memory reconsolidation impairs drug reward memory. However, the underlying mechanisms remain elusive. Although actin polymerization is involved in memory formation, its role in the reconsolidation of drug reward memory is unknown. In addition, the specific brain areas responsible for drug memory have not been fully identified. In the present study, we found that inhibiting actin polymerization in the nucleus accumbens (NAc) shell, but not the NAc core, abolishes morphine-induced conditioned place preference (CPP) by disrupting its reconsolidation in rats. Moreover, this effect persists for more than 2 weeks by a single injection of the actin polymerization inhibitor, which is not reversed by a morphine-priming injection. Furthermore, the application of actin polymerization inhibitor outside the reconsolidation window has no effect on morphine-associated contextual memory. Taken together, our findings first demonstrate that inhibiting actin polymerization erases morphine-induced CPP by disrupting its reconsolidation. Our study suggests that inhibition of actin polymerization during drug memory reconsolidation may be a potential approach to prevent drug relapse.

Drug addiction is a chronic brain disorder with a high rate of relapse, which is characterized by compulsive drug seeking and drug taking despite harmful consequences^1^,^2^. It not only increases health care costs but also causes enormous social problems, for example, increased rates of disability and crime. However, even after prolonged abstinence, high relapse rates limit successful treatment of drug addiction^3^,^4^,^5^. The major cause of relapse is the robust and long-lasting memory formed by learned association between rewarding or aversive drug effects and drug-related environmental cues^6^,^7^. Thus, it is urgent to develop a therapeutic approach to disrupt the associative memory between drug and drug-related cues. Memory reconsolidation sheds light on blocking this association to prevent contextual cue-induced relapse.

After learning, new memories are stabilized, named memory consolidation^8^. Then, retrieval of a memory trace induces an additional labile phase that requires an active process to restabilize the established memory, which is named reconsolidation^9^,^10^,^11^,^12^,^13^,^14^. Consistently, a short labile phase can be induced once consolidated drug-associated memories are retrieved, which provides a window to manipulate the established drug-associated memory.

Although underlying mechanisms of drug memory reconsolidation remain elusive, increased evidence suggests that dyregulation of actin dynamics may play a key role in this process. First, actin is the primary structural component of cells and highly dynamic conversion between actin monomers (G-actin) and branched filaments (F-actin), i.e., actin rearrangements^15^, is essential to maintain cellular functions, particularly in neurons^16^,^17^. Second, actin dynamics plays a crucial role in memory formation. For example, inhibition of actin polymerization by latrunculin A (Lat A) disrupts the late phase of long-term potentiation^18^. Rehberg *et al.* showed that actin filament arrest in the basolateral complex of the amygdala impairs fear memory consolidation and reconsolidation^19^. In addition, actin rearrangement is involved in drug-related memories^20^,^21^,^22^. Moreover, systemic administration of rapamycin, an inhibitor of mTOR kinase, blocks morphine-induced CPP after re-exposure to morphine-paired environment cues, while rapamycin could inhibit actin reorganization^23^. Above evidence suggests that actin dynamics may be involved in the reconsolidation of drug memories, e.g., morphine-induced CPP.

Nucleus accumbens (NAc) is a central component of the limbic system, which plays a major role in reward and addiction^24^. The NAc consists of two main substructures, the core and shell, which are the inner and outer region of the NAc, respectively. Both of them are involved in reward and learning reinforcement. Previous studies suggest that they not only have distinct roles but also have overlap roles to certain extent in the above processes. First, reinstatement of drug-seeking behavior induced by re-exposure to heroin-associated environment cues or acute food deprivation stress requires the activation of D1-like receptors in the NAc shell but not that in the core^25^,^26^. On the other hand, infusion of lactacystin, a proteasome inhibitor, into the core, but not the shell, prevents cocaine-associated memory extinction^27^. Interestingly, microinjections of activity-regulated cytoskeleton-associated protein (Arc/Arg3.1) antisense oligodeoxynucleotides into the NAc core impair the acquisition, expression and reinstatement of morphine-induced CPP, while intra-NAc shell injections only impair the expression of morphine-induced CPP^28^. However, the role of the NAc core and shell in the reconsolidation of morphine-induced CPP and underlying mechanisms remain unknown.

In the present study, we report that inhibiting actin polymerization in the NAc shell, but not the NAc core, abolishes morphine-induced CPP by disrupting its reconsolidation. Moreover, this effect persists for more than 2 weeks by a single injection of the actin polymerization inhibitor, which is not reversed by a morphine-priming injection. Furthermore, the application of actin polymerization inhibitor outside the reconsolidation window has no effect on morphine-associated contextual memory. Our study suggests that inhibition of actin polymerization during drug memory reconsolidation may be a potential approach to prevent drug relapse.

## Results

### Inhibition of actin polymerization in the NAc core has no effect on the expression of morphine-induced CPP

**Figure 1:**
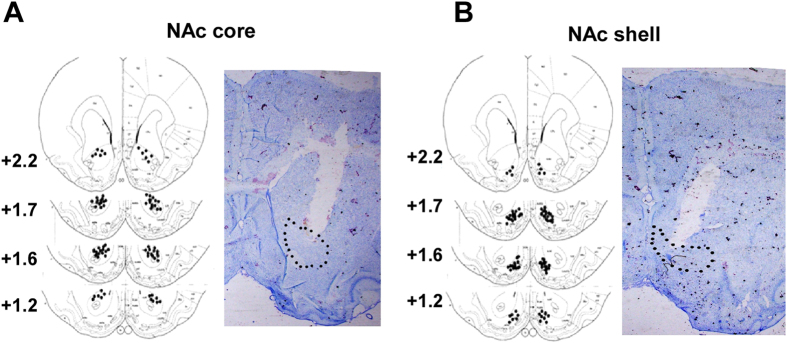
Schematic representation and photomicrographs of microinjection sites. (**A**) Distribution of microinjection sites in the NAc core (top panel) and the photomicrographs of representative sample punches and cannula placements in the NAc core (bottom panel). (**B**) Distribution of microinjection sites in the NAc shell (top panel) and the photomicrographs of representative sample punches and cannula placements in the NAc shell (bottom panel).

**Figure 2:**
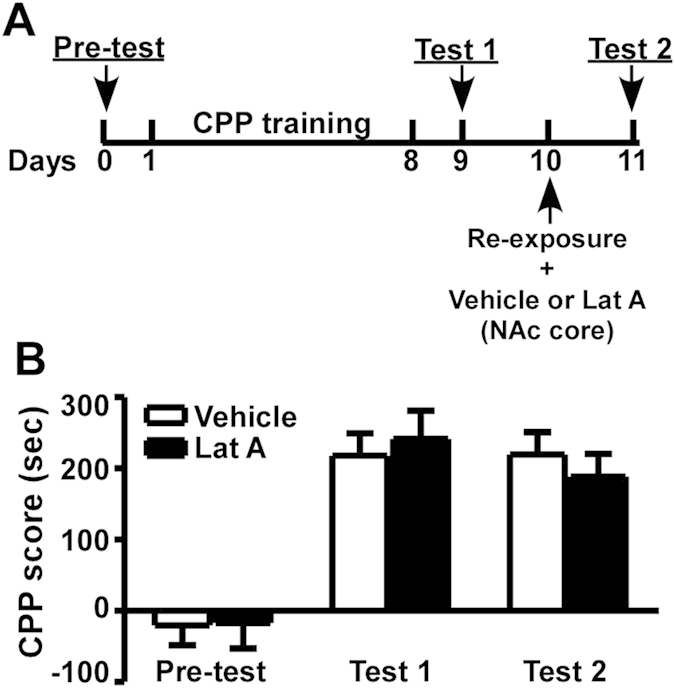
Inhibition of actin polymerization in the NAc core has no effect on the expression of morphine-induced CPP. (**A**) Timeline of the experimental procedure. (**B**) CPP scores of rats receiving microinjections into the NAc core. Values represent mean ± SEM, n = 7–8/group.

After a pre-test of CPP, all rats received 8 days of CPP training, one session per day. The first CPP test was performed on day 9. On day 10, the rats received memory retrieval (i.e., re-exposure to the morphine-paired chamber for 10 minute) immediately followed by microinjections of vehicle or Lat A (an actin polymerization inhibitor) into the NAc core at the concentration of 0.5 μg/μl. On the next day, the CPP test (test 2) was performed (Figs 1A and 2A). Statistical analysis of CPP scores of rats receiving vehicle or Lat A microinjections revealed a significant effect of test phase (F_2,26_ = 34.915, P < 0.01), while no significant effect was found between vehicle and Lat A treatments (P > 0.05) as well as from the interaction between treatment and test phase (P > 0.05) (Fig. 2B). These results demonstrate that inhibition of actin polymerization in the NAc core has no effect on the expression of morphine-induced CPP.

### Inhibition of actin polymerization in the NAc shell inhibits morphine-induced CPP by disrupting its reconsolidation

**Figure 3:**
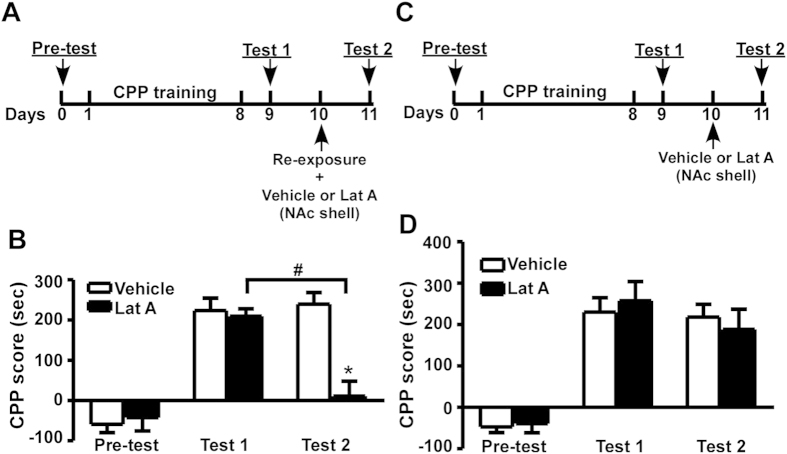
Inhibition of actin polymerization in the NAc shell inhibits morphine-induced CPP by disrupting its reconsolidation. (**A**) Timeline of the experimental procedure. (**B**) CPP scores of rats receiving microinjections into the NAc shell. (**C**) Timeline of the experimental procedure. Compared with (**A**), no re-exposure to the morphine-paired chamber was performed on day 9. (**D**) CPP scores of rats receiving microinjections into the NAc shell without re-exposure to the morphine-paired chamber. Values represent mean ± SEM, n = 7–8/group. *P < 0.01 vs. test 2 of the vehicle group and ^#^P < 0.01 vs. test 1 of the Lat A group, two-way ANOVA with Tukey’s post hoc test.

To further investigate the effect of inhibition of actin polymerization in the NAc shell, the rats immediately received microinjections of vehicle or Lat A into the NAc shell instead of the NAc core after re-exposure to the morphine-paired chamber on day 10 (Figs 1B and 3A). A significant effect of test phase (F_2,26_ = 32.646, P < 0.01), treatment (F_1,13_ = 17.905, P < 0.01) and treatment × test phase interaction (F_2,26_ = 6.810, P < 0.01) was revealed (Fig. 3B). Moreover, Tukey’s post hoc analysis showed that a single Lat A injection completely inhibited the expression of morphine-induced CPP compared with vehicle treatment, 11.06 ± 37.24 s vs. 239.51 ± 28.55 s, p < 0.01 (Fig. 3B). These results indicate that a single Lat A microinjection into the shell inhibits the expression of morphine-induced CPP.

To investigate whether Lat A-induced disappearance of CPP expression is mediated by the disruption of its reconsolidation, the experiments without retrieval were performed as retrieval is an essential step of memory reconsolidation (Fig. 3C). The first 9-day procedure is as same as that shown in Fig. 3A. On day 10, the rats received microinjections of vehicle or Lat A into the NAc shell without re-exposure to the morphine-paired chamber. On day 11, the CPP test (test 2) was performed. As expected, Lat A had no significant effect on the expression of morphine-induced CPP compared with vehicle treatment (P > 0.05), although a significant effect of test phase (F_2,24_ = 35.960, P < 0.01) was maintained. In addition, the treatment × test phase interaction was not significant (F_2,24_ = 0.311, p > 0.05) (Fig. 3D). These results demonstrate that retrieval is essential for Lat A-induced disappearance of CPP, indicating that the disruption of memory reconsolidation by Lat A contributes to the disappearance of CPP expression.

### Inhibition of actin polymerization in the NAc shell has a long-term effect on disrupting morphine-induced CPP

**Figure 4:**
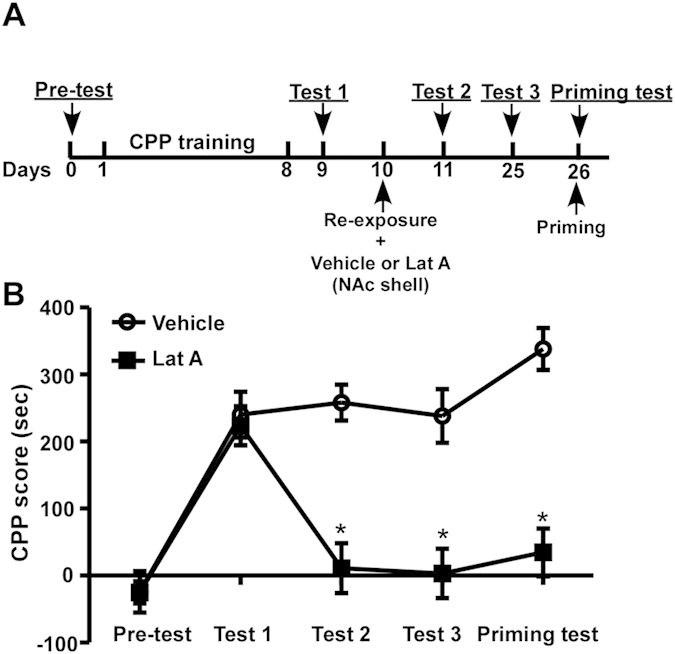
Inhibition of actin polymerization in the NAc shell has a long-term effect on disrupting morphine-induced CPP. (**A**) Timeline of the experimental procedure. (**B**) CPP scores of rats receiving microinjections into the NAc shell. Values represent mean ± SEM, n = 7–8/group. *P < 0.01 vs. each corresponding test of the vehicle group, two-way ANOVA with Tukey’s post hoc test.

We have shown that a single post-retrieval microinjection of Lat A inhibited the expression of morphine-induced CPP (Fig. 3B). To further examine the long-term effect of Lat A treatment, morphine-induced CPP was re-tested after two weeks (test 3) (Fig. 4A). On day 26, the priming test of CPP was performed immediately after a priming injection of morphine. Statistical analysis revealed a significant effect of test phase (F_4,52_ = 17.028, P < 0.01), treatment (F_1,13_ = 58.597, P < 0.01) and treatment × test phase interaction (F_4,52_ = 9.012, P < 0.01). More importantly, Lat A-mediated disappearance of morphine-induced CPP was persistent for more than 2 weeks compared with vehicle treatment, 3.29 ± 36.95 s vs. 238.06 ± 40.00 s (test 3), P < 0.01 by Tukey’s post hoc test, and the morphine injection did not induce the reinstatement of morphine-induced CPP in Lat A-treated rats, 34.67 ± 35.52 s vs. 338.2 ± 31.15 s, P < 0.01 by Tukey’s post hoc test (Fig. 4B). These results indicate that a single Lat A microinjection has a persistent effect on the disruption of morphine-induced CPP for more than 2 weeks.

### Inhibition of actin polymerization in the NAc shell outside the reconsolidation window has no effect on morphine-induced CPP

**Figure 5:**
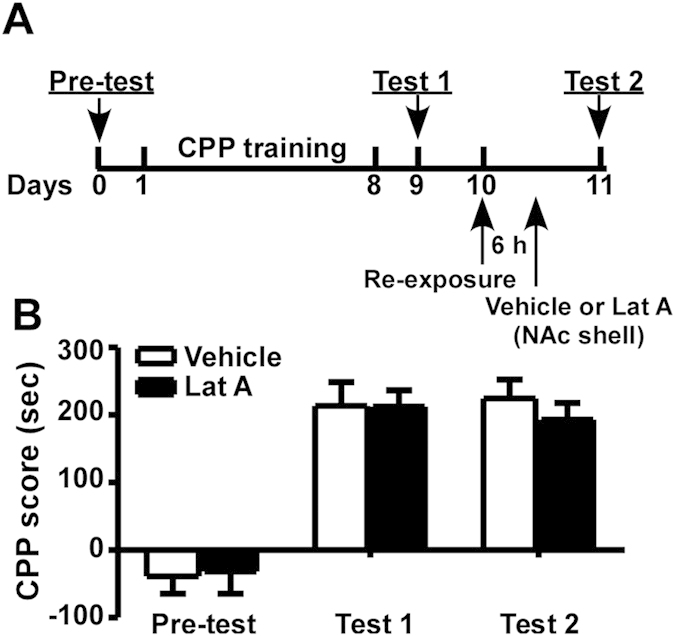
Inhibition of actin polymerization in the NAc shell outside the reconsolidation window has no effect on morphine-induced CPP. (**A**) Timeline of the experimental procedure. On day 10, the rats received vehicle or Lat A microinjections 6 hours after re-exposure to the morphine-paired chamber. (**B**) CPP scores of rats receiving microinjections into the NAc shell. Values represent mean ± SEM, n = 7–8/group.

Previous studies showed that the time course of memory reconsolidation ranges from minutes to hours. However, the time window of morphine-induced CPP reconsolidation is unknown, which is essential for clinical applications. To further confirm that inhibition of actin polymerization in the NAc shell inhibits morphine-induced CPP by disrupting its reconsolidation and test the window of reconsolidation of morphine-induced CPP, the microinjection of Lat A was performed 6 hours after retrieval and morphine-induced CPP was tested on the next day (test 2) (Fig. 5A). Although statistical analysis of CPP scores revealed a significant effect of test phase (F_2,24_ = 33.847, P < 0.01), no significant difference between vehicle and Lat A treatments was detected (F_1,12_ = 0.483, P > 0.05) and no significant difference was revealed from the interaction between treatment and test phase (P > 0.05; Fig. 5B). These results demonstrate that Lat A treatment applied 6 hours after retrieval, which is outside the reconsolidation window, has no effect on the expression of morphine-induced CPP.

## Discussion

Relapse is a major challenge of drug addiction recovery. Even after prolonged abstinence, re-exposure to drug-related environmental cues can re-activate drug reward memory and elicit drug craving, leading to reinstatement of drug-seeking behavior, which is a major cause of failure in preventing relapse. Thus, it is necessary to develop a therapeutic approach to disrupt the associative memory between drug and drug-related cues. Recent studies suggest that memory reconsolidation plays a key role in contextual cue-induced relapse^29^,^30^,^31^,^32^,^33^,^34^,^35^,^36^. In addition, actin dynamics is implicated in memory consolidation and reconsolidation^19^. Moreover, accumulating evidence suggests that actin dynamics is implicated in drug addiction and reinstatement. For example, actin rearrangement is involved in drug-associated memories^20^,^21^. Glutamate receptors, calcium/calmodulin-dependent protein kinase II (CAMKII), mammalian target of rapamycin (mTOR), Lim-kinase (LIMK) and Rho guanosine triphosphatases are involved in drug addiction and its treatment, which all negatively or positively affect actin rearrangements^37^,^38^,^39^,^40^,^41^,^42^. Although the evidence suggests that manipulating actin dynamics may be a potential approach to disrupt cue-associated drug memories by affecting memory reconsolidation, no direct evidence confirm this hypothesis. In this study, we demonstrate that inhibiting actin polymerization disrupts morphine-induce CPP, mediated by disrupting the reconsolidation of associative memories between morphine and morphine-related cues. In addition, a single post-retrieval injection of Lat A has robust and long-term benefits to inhibit morphine-induced CPP, indicating that post-retrieval inhibition of actin polymerization is a potential approach to prevent relapse, which has a long lasting effect.

Although both the NAc shell and core are involved in drug addiction and drug memory reconsolidation, they do have distinct functions in these processes. Previous studies showed that the NAc core play an important role in drug memory reconsolidation. For example, infusion of the protein synthesis inhibitor into the NAc core disrupts the reconsolidation of cocaine-associated memory^31^. Recently, it has been reported that NAc shell Arc/Arg3.1 protein, but not the protein in the NAc core, mediates the reconsolidation of morphine-induced CPP^43^. Our data first showed that disrupting actin polymerization in the NAc shell contributes to the impaired reconsolidation of morphine-induced CPP, while the NAc core is not involved in this process. The above evidence suggests that the NAc core and shell play distinct roles in drug memory reconsolidation depending on differential drugs (e.g., cocaine and morphine) and molecular pathways (e.g., protein synthesis, proteasome degradation and actin dynamics) involved.

Our data indicates that inhibiting actin polymerization in the NAc shell inhibits morphine-induce CPP mediated by disrupting drug memory reconsolidation. Young *et al.* showed that inhibiting actin polymerization in the basolateral amygdala (BLA) inhibits the consolidation of the methamphetamine-associated memory, but not the reconsolidation^20^. It seems like that the effect of Lat A, the actin polymerization inhibitor, on drug memory is contradictory. However, two key points should be noted, different brain regions and different drugs. First, the NAc shell and BLA play differential roles in memory process, which may contribute to differential effects of Lat A on the memory formation, storage and maintenance^44^. For example, the BLA is mainly involved in emotional modulation of memory, while the NAc plays a major role in drug reward memory^31^,^43^,^45^,^46^,^47^,^48^. In addition, morphine and methamphetamine induce drug addiction through differential mechanisms, which may also contribute to differential effects of actin polymerization inhibition. For example, morphine indirectly induces dopamine release mediated by opioid receptor activation, while methamphetamine directly increases dopamine level by inhibiting dopamine transporter-mediated dopamine reuptake and potentiating dopamine release^43^,^49^,^50^,^51^,^52^,^53^.

In conclusion, we first demonstrate that actin polymerization in the NAc shell plays a pivotal role in the reconsolidation of morphine-induced CPP. Moreover, inhibition of actin polymerization abolishes morphine-induced CPP by disrupting its reconsolidation. Our work provides a novel insight that inhibition of actin polymerization is a potential approach to prevent relapse and the intervention should be applied within a limited time window after retrieval.

## Materials and Methods

### Subjects

Male Sprague-Dawley rats, weighing 230–250 g, were obtained from the Laboratory Animal Center, Peking University Health Science Center. They were housed four per cage in an animal facility with constant temperature (23 ± 2 °C) and humidity (50 ± 5%). The rats access to water and food ad libitum under a reverse 12 h light: 12 h dark cycle. The experimental procedures were carried out in accordance with the National Institutes of Health Guide for the Care and Use of Laboratory Animals, and were approved by the Local Animal Care and Use Committee.

### Drugs

Morphine sulfate (Qinghai Pharmaceutical Ltd, Xining, China) was dissolved in 0.9% physiological saline and injected subcutaneously (10 mg/kg) in a volume of 1 ml/kg before exposure to a drug-paired context during the CPP training sessions. Latrunculin A (Lat A) (Merck, 428021-100UG, Germany) was dissolved in DMSO (25 μg/μl) and diluted in PBS to a final concentration of 0.5 μg/μl. The dose of Lat A was chosen based on the work by Hou *et al.*^54^. The vehicle was PBS which contained 2% DMSO.

### Surgery and intracranial injections

All surgical procedures were performed as previously described^55^. Briefly, rats weighing 290–310 g were anesthetized with sodium pentobarbital (60 mg/kg, i.p.), and permanent guide cannulae (23 gauge; Plastics One, Roanoke, VA, USA) were implanted bilaterally 1 mm above the NAc shell and core. The stereotaxic coordinates for the NAc shell were anterior/posterior (AP), +1.8 mm, medial/lateral (ML), +3.2 mm (16° angle) and dorsal/ventral (DV), −6.6 mm. The stereotaxic coordinates for the NAc core were AP, +1.5 mm, ML, +3.8 mm (16° angle) and DV, −6.0 mm. Stainless steel screws and dental cement were used to anchor the cannulae to the skull. Each cannula was inserted by a stainless steel blocker to maintain patency and prevent infection. The rats were given 1 week to recover from surgery before the subsequent experiments were carried out.

Injections were carried out with Hamilton microsyringes, which were connected to 30-gauge injectors (Plastics One,USA). Lat A (0.5 μg/μl/side) or vehicle (0.5 ul/side) was infused bilaterally into the NAc core and shell (Fig. 1), respectively, at a rate of 0.5 μl/min. The injectors were kept in place for another 2 minutes to allow the drug to completely diffuse to the target region.

### Conditioned place preference (CPP)

Morphine-induced CPP was carried out using an unbiased, counterbalanced protocol. CPP training procedures were described previously^25^,^56^. Briefly, the CPP apparatus consists of nine identical three-chamber polyvinyl chloride (PVC) boxes. In each box, two large chambers (27.9 cm long × 21.0 cm wide × 20.9 cm high) are separated by a smaller chamber (12.1 cm long × 21.0 cm wide × 20.9 cm high). The three distinct chambers were divided by manual guillotine doors. The floor texture of the two large chambers (bar or grid, respectively) was different from each other to provide distinct visual cues paired with morphine or saline injections.

In the baseline place preference test (preconditioning test, pre-test), the rats were initially placed in the center chamber and allowed ad libitum access to the three chambers for 15 minutes. The time spent in the designated morphine- or saline-paired chamber during the 15-minute session was recorded by detecting infrared beam breaks. Twenty-one rats showing a strong unconditioned preference (>540 s) for one compartment were excluded. During the conditioning days, the rats were trained for 8 consecutive days with alternative injections of morphine (10 mg/ml/kg, s.c.) or saline (1 ml/kg, s.c.) in the designated chambers. After each injection, the rats were placed in the morphine- or saline-conditioned chamber for 45 minutes before brought back to their home cages. One day after the last conditioning trial, the expression of morphine-induced CPP was tested under conditions identical to those described in the baseline preference test. The CPP score was defined as the difference in time (in seconds) spent in the morphine-paired and saline-paired chambers during CPP testing.

### Experimental design

Experiment 1: Two groups of rats were (n = 7–8 per group) trained for morphine-induced CPP for 8 days. On day 9, all rats were tested for morphine-induce CPP (test 1). On day 10, all rats were re-exposed to the morphine-paired chamber for 10 minutes to reactivate morphine-associated memory, referred as reactivation or retrieval. Two groups of rats received bilateral Lat A (0.5 μg/μl) or vehicle (0.5 μl) microinjections into the NAc core immediately after the reactivation, respectively. On day 11, all rats were retested for morphine-induced CPP (test 2).

Experiment 2: Four groups of rats (n = 7–8 per group) were trained for morphine-induced CPP for 8 days. On day 9, all rats were tested for morphine-induce CPP (test 1). On day 10, two groups of rats were re-exposed to the morphine-paired chamber for 10 minutes and received bilateral Lat A (0.5 μg/μl) or vehicle (0.5 μl) microinjections into the NAc shell immediately after the reactivation. In the meantime, the other two groups of rats without reactivation received bilateral Lat A (0.5 μg/μl) or vehicle (0.5 μl) microinjections into the NAc shell, respectively. On day 11, all four rats were retested for morphine-induced CPP (test 2).

Experiment 3: Two groups of rats (n = 7–8 per group) were trained for morphine-induced CPP for 8 days. On day 9, all rats were tested for morphine-induce CPP (test 1). On day 10, two groups of rats were re-exposed to the morphine-paired chamber for 10 minutes and received bilateral Lat A (0.5 μg/μl) or vehicle (0.5 μl) microinjections into the NAc shell immediately after the reactivation. On day 11 and 25, all rats were retested for morphine-induced CPP (test 2 and test 3). On day 26, both vehicle and Lat A-treated rats received a priming injection of morphine (3 mg/ml/kg, s.c.), and the CPP test was immediately performed after the priming injection (priming test).

Experiment 4: Two groups of rats (n = 7–8 per group) were trained for morphine-induced CPP for 8 days. On day 9, all rats were tested for morphine-induce CPP (test 1). On day 10, two groups of rats were re-exposed to the morphine-paired chamber for 10 minutes and received bilateral Lat A (0.5 μg/μl) or vehicle (0.5 μl) microinjections into the NAc shell 6 hours after the reactivation. On day 11, all rats were retested for morphine-induced CPP (test 2).

### Statistical analysis

Values are expressed as mean ± SEM. The data were analyzed by two-way repeated-measures analysis of variance (ANOVA) with the between-subjects factor of treatment (vehicle or Lat A) and within-subjects factor of test phase, followed by Tukey’s post hoc test. p < 0.05 was accepted as statistical significance.

## Additional Information

**How to cite this article**: Li, G. *et al.* Inhibition of actin polymerization in the NAc shell inhibits morphine-induced CPP by disrupting its reconsolidation. *Sci. Rep.* **5**, 16283; doi: 10.1038/srep16283 (2015).

## References

[b1] O’Brien C. P. & McLellan A. T. Myths about the treatment of addiction. Lancet 347, 237–40 (1996). 8551886 10.1016/s0140-6736(96)90409-2

[b2] Leshner A. I. Addiction is a brain disease, and it matters. Science 278, 45–7 (1997). 9311924 10.1126/science.278.5335.45

[b3] Grimm J. W., Hope B. T., Wise R. A. & Shaham Y. Neuroadaptation. Incubation of cocaine craving after withdrawal. Nature 412, 141–2 (2001). 11449260 10.1038/35084134 PMC2889613

[b4] Lu L. *et al.* Central amygdala ERK signaling pathway is critical to incubation of cocaine craving. Nat Neurosci 8, 212–9 (2005). 15657599 10.1038/nn1383

[b5] Millan E. Z., Milligan-Saville J. & McNally G. P. Memory retrieval, extinction, and reinstatement of alcohol seeking. Neurobiol Learn Mem 101, 26–32 (2013). 23305621 10.1016/j.nlm.2012.12.010

[b6] Hyman S. E., Malenka R. C. & Nestler E. J. Neural mechanisms of addiction: the role of reward-related learning and memory. Annu Rev Neurosci 29, 565–98 (2006). 16776597 10.1146/annurev.neuro.29.051605.113009

[b7] Robbins T. W., Ersche K. D. & Everitt B. J. Drug addiction and the memory systems of the brain. Ann N Y Acad Sci 1141, 1–21 (2008). 18991949 10.1196/annals.1441.020

[b8] McGaugh J. L. Memory--a century of consolidation. Science 287, 248–51 (2000). 10634773 10.1126/science.287.5451.248

[b9] Nestler E. J. Neurobiology. Total recall-the memory of addiction. Science 292, 2266–7 (2001). 11423644 10.1126/science.1063024

[b10] Tronson N. C., Wiseman S. L., Olausson P. & Taylor J. R. Bidirectional behavioral plasticity of memory reconsolidation depends on amygdalar protein kinase A. Nat Neurosci 9, 167–9 (2006). 16415868 10.1038/nn1628

[b11] Tronson N. C. & Taylor J. R. Molecular mechanisms of memory reconsolidation. Nat Rev Neurosci 8, 262–75 (2007). 17342174 10.1038/nrn2090

[b12] Li F. Q. *et al.* Basolateral amygdala cdk5 activity mediates consolidation and reconsolidation of memories for cocaine cues. J Neurosci 30, 10351–9 (2010). 20685978 10.1523/JNEUROSCI.2112-10.2010 PMC3150196

[b13] Nader K., Schafe G. E. & Le Doux J. E. Fear memories require protein synthesis in the amygdala for reconsolidation after retrieval. Nature 406, 722–6 (2000). 10963596 10.1038/35021052

[b14] Dudai Y. Reconsolidation: the advantage of being refocused. Curr Opin Neurobiol 16, 174–8 (2006). 16563730 10.1016/j.conb.2006.03.010

[b15] Star E. N., Kwiatkowski D. J. & Murthy V. N. Rapid turnover of actin in dendritic spines and its regulation by activity. Nat Neurosci 5, 239–46 (2002). 11850630 10.1038/nn811

[b16] Cingolani L. A. & Goda Y. Actin in action: the interplay between the actin cytoskeleton and synaptic efficacy. Nat Rev Neurosci 9, 344–56 (2008). 18425089 10.1038/nrn2373

[b17] Kasai H., Fukuda M., Watanabe S., Hayashi-Takagi A. & Noguchi J. Structural dynamics of dendritic spines in memory and cognition. Trends Neurosci 33, 121–9 (2010). 20138375 10.1016/j.tins.2010.01.001

[b18] Fukazawa Y. *et al.* Hippocampal LTP is accompanied by enhanced F-actin content within the dendritic spine that is essential for late LTP maintenance *in vivo*. Neuron 38, 447–60 (2003). 12741991 10.1016/s0896-6273(03)00206-x

[b19] Rehberg K., Bergado-Acosta J. R., Koch J. C. & Stork O. Disruption of fear memory consolidation and reconsolidation by actin filament arrest in the basolateral amygdala. Neurobiol Learn Mem 94, 117–26 (2010). 20416387 10.1016/j.nlm.2010.04.007

[b20] Young E. J. *et al.* Selective, retrieval-independent disruption of methamphetamine-associated memory by actin depolymerization. Biol Psychiatry 75, 96–104 (2014). 24012327 10.1016/j.biopsych.2013.07.036 PMC4023488

[b21] Young E. J., Briggs S. B. & Miller C. A. The Actin Cytoskeleton as a Therapeutic Target for the Prevention of Relapse to Methamphetamine Use. CNS Neurol Disord Drug Targets 14, 731–7 (2015). 26022262 10.2174/1871527314666150529145531 PMC4641563

[b22] Young E. J. *et al.* Nonmuscle myosin IIB as a therapeutic target for the prevention of relapse to methamphetamine use. Mol Psychiatry (2015). 10.1038/mp.2015.103 PMC4740255 26239291

[b23] Lin J. *et al.* Rapamycin prevents drug seeking via disrupting reconsolidation of reward memory in rats. Int J Neuropsychopharmacol 17, 127–36 (2014). 24103337 10.1017/S1461145713001156

[b24] Heimer L., Zahm D. S., Churchill L., Kalivas P. W. & Wohltmann C. Specificity in the projection patterns of accumbal core and shell in the rat. Neuroscience 41, 89–125 (1991). 2057066 10.1016/0306-4522(91)90202-y

[b25] Bossert J. M., Poles G. C., Wihbey K. A., Koya E. & Shaham Y. Differential effects of blockade of dopamine D1-family receptors in nucleus accumbens core or shell on reinstatement of heroin seeking induced by contextual and discrete cues. J Neurosci 27, 12655–63 (2007). 18003845 10.1523/JNEUROSCI.3926-07.2007 PMC2117350

[b26] Tobin S., Sedki F., Abbas Z. & Shalev U. Antagonism of the dopamine D1-like receptor in mesocorticolimbic nuclei attenuates acute food deprivation-induced reinstatement of heroin seeking in rats. Eur J Neurosci 37, 972–81 (2013). 23320810 10.1111/ejn.12112

[b27] Ren Z. Y. *et al.* A critical role for protein degradation in the nucleus accumbens core in cocaine reward memory. Neuropsychopharmacology 38, 778–90 (2013). 23303053 10.1038/npp.2012.243 PMC3672001

[b28] Lv X. F., Xu Y., Han J. S. & Cui C. L. Expression of activity-regulated cytoskeleton-associated protein (Arc/Arg3.1) in the nucleus accumbens is critical for the acquisition, expression and reinstatement of morphine-induced conditioned place preference. Behav Brain Res 223, 182–91 (2011). 21549764 10.1016/j.bbr.2011.04.029

[b29] Lattal K. M. & Abel T. Behavioral impairments caused by injections of the protein synthesis inhibitor anisomycin after contextual retrieval reverse with time. Proc Natl Acad Sci USA 101, 4667–72 (2004). 15070775 10.1073/pnas.0306546101 PMC384804

[b30] Bernardi R. E., Lattal K. M. & Berger S. P. Postretrieval propranolol disrupts a cocaine conditioned place preference. Neuroreport 17, 1443–7 (2006). 16932155 10.1097/01.wnr.0000233098.20655.26

[b31] Valjent E., Corbille A. G., Bertran-Gonzalez J., Herve D. & Girault J. A. Inhibition of ERK pathway or protein synthesis during reexposure to drugs of abuse erases previously learned place preference. Proc Natl Acad Sci USA 103, 2932–7 (2006). 16473939 10.1073/pnas.0511030103 PMC1413817

[b32] Fricks-Gleason A. N. & Marshall J. F. Post-retrieval beta-adrenergic receptor blockade: effects on extinction and reconsolidation of cocaine-cue memories. Learn Mem 15, 643–8 (2008). 18772251 10.1101/lm.1054608 PMC2632789

[b33] Robinson M. J. & Franklin K. B. Reconsolidation of a morphine place preference: impact of the strength and age of memory on disruption by propranolol and midazolam. Behav Brain Res 213, 201–7 (2010). 20457186 10.1016/j.bbr.2010.04.056

[b34] Wu Y., Li Y., Gao J. & Sui N. Differential effect of NMDA receptor antagonist in the nucleus accumbens on reconsolidation of morphine -related positive and aversive memory in rats. Eur J Pharmacol 674, 321–6 (2012). 22119382 10.1016/j.ejphar.2011.11.011

[b35] Wells A. M. *et al.* Extracellular signal-regulated kinase in the basolateral amygdala, but not the nucleus accumbens core, is critical for context-response-cocaine memory reconsolidation in rats. Neuropsychopharmacology 38, 753–62 (2013). 23232446 10.1038/npp.2012.238 PMC3671999

[b36] Wu Y., Li Y., Yang X. & Sui N. Differential effect of beta-adrenergic receptor antagonism in basolateral amygdala on reconsolidation of aversive and appetitive memories associated with morphine in rats. Addict Biol 19, 5–15 (2014). 22458530 10.1111/j.1369-1600.2012.00443.x

[b37] Fischer M., Kaech S., Wagner U., Brinkhaus H. & Matus A. Glutamate receptors regulate actin-based plasticity in dendritic spines. Nat Neurosci 3, 887–94 (2000). 10966619 10.1038/78791

[b38] Hoffman L., Farley M. M. & Waxham M. N. Calcium-calmodulin-dependent protein kinase II isoforms differentially impact the dynamics and structure of the actin cytoskeleton. Biochemistry 52, 1198–207 (2013). 23343535 10.1021/bi3016586 PMC3578116

[b39] Huang W. *et al.* mTORC2 controls actin polymerization required for consolidation of long-term memory. Nat Neurosci 16, 441–8 (2013). 23455608 10.1038/nn.3351 PMC3615448

[b40] Meng Y., Zhang Y., Tregoubov V., Falls D. L. & Jia Z. Regulation of spine morphology and synaptic function by LIMK and the actin cytoskeleton. Rev Neurosci 14, 233–40 (2003). 14513866 10.1515/revneuro.2003.14.3.233

[b41] Nobes C. D. & Hall A. Rho, rac, and cdc42 GTPases regulate the assembly of multimolecular focal complexes associated with actin stress fibers, lamellipodia, and filopodia. Cell 81, 53–62 (1995). 7536630 10.1016/0092-8674(95)90370-4

[b42] Ridley A. J., Paterson H. F., Johnston C. L., Diekmann D. & Hall A. The small GTP-binding protein rac regulates growth factor-induced membrane ruffling. Cell 70, 401–10 (1992). 1643658 10.1016/0092-8674(92)90164-8

[b43] Lv X. F., Sun L. L., Cui C. L. & Han J. S. NAc Shell Arc/Arg3.1 Protein Mediates Reconsolidation of Morphine CPP by Increased GluR1 Cell Surface Expression: Activation of ERK-Coupled CREB is Required. Int J Neuropsychopharmacol (2015). 10.1093/ijnp/pyv030 PMC4576513 25746394

[b44] Ding Z. B. *et al.* Region-specific role of Rac in nucleus accumbens core and basolateral amygdala in consolidation and reconsolidation of cocaine-associated cue memory in rats. Psychopharmacology (Berl) 228, 427–37 (2013). 23494234 10.1007/s00213-013-3050-8

[b45] Di Chiara G. Nucleus accumbens shell and core dopamine: differential role in behavior and addiction. Behav Brain Res 137, 75–114 (2002). 12445717 10.1016/s0166-4328(02)00286-3

[b46] Fuchs R. A., Evans K. A., Parker M. C. & See R. E. Differential involvement of the core and shell subregions of the nucleus accumbens in conditioned cue-induced reinstatement of cocaine seeking in rats. Psychopharmacology (Berl) 176, 459–65 (2004). 15138757 10.1007/s00213-004-1895-6

[b47] McGaugh J. L. The amygdala modulates the consolidation of memories of emotionally arousing experiences. Annu Rev Neurosci 27, 1–28 (2004). 15217324 10.1146/annurev.neuro.27.070203.144157

[b48] Richter-Levin G. The amygdala, the hippocampus, and emotional modulation of memory. Neuroscientist 10, 31–9 (2004). 14987446 10.1177/1073858403259955

[b49] Johnson S. W. & North R. A. Opioids excite dopamine neurons by hyperpolarization of local interneurons. J Neurosci 12, 483–8 (1992). 1346804 10.1523/JNEUROSCI.12-02-00483.1992 PMC6575608

[b50] Jalabert M. *et al.* Neuronal circuits underlying acute morphine action on dopamine neurons. Proc Natl Acad Sci USA 108, 16446–50 (2011). 21930931 10.1073/pnas.1105418108 PMC3182694

[b51] Fleckenstein A. E., Volz T. J., Riddle E. L., Gibb J. W. & Hanson G. R. New insights into the mechanism of action of amphetamines. Annu Rev Pharmacol Toxicol 47, 681–98 (2007). 17209801 10.1146/annurev.pharmtox.47.120505.105140

[b52] Sulzer D., Sonders M. S., Poulsen N. W. & Galli A. Mechanisms of neurotransmitter release by amphetamines: a review. Prog Neurobiol 75, 406–33 (2005). 15955613 10.1016/j.pneurobio.2005.04.003

[b53] Rothman R. B. & Baumann M. H. Monoamine transporters and psychostimulant drugs. Eur J Pharmacol 479, 23–40 (2003). 14612135 10.1016/j.ejphar.2003.08.054

[b54] Hou Y. Y. *et al.* Involvement of actin rearrangements within the amygdala and the dorsal hippocampus in aversive memories of drug withdrawal in acute morphine-dependent rats. J Neurosci 29, 12244–54 (2009). 19793983 10.1523/JNEUROSCI.1970-09.2009 PMC6666133

[b55] Xu C. M. *et al.* Glycogen synthase kinase 3beta in the nucleus accumbens core mediates cocaine-induced behavioral sensitization. J Neurochem 111, 1357–68 (2009). 19799712 10.1111/j.1471-4159.2009.06414.x

[b56] Zhou S. J. *et al.* NMDA receptor glycine modulatory site in the ventral tegmental area regulates the acquisition, retrieval, and reconsolidation of cocaine reward memory. Psychopharmacology (Berl) 221, 79–89 (2012). 22105219 10.1007/s00213-011-2551-6

